# UDHAVI Community Support During India's Second COVID-19 Wave: A Descriptive Study on a Tertiary Care Center's Pandemic Response Helpline

**DOI:** 10.9745/GHSP-D-22-00315

**Published:** 2023-10-30

**Authors:** Balu Krishna Sasidharan, Ranjit Immanuel James, Sowmya Sathyendra, R. Harsh, Jenifer Jeba Sundararaj, Vinitha Ravindran, Hannah Mary Thomas T, Narmada Ashok, Madan Mohan Thirunavukkarasu, John Victor Punitha, Tarun K. George, Barney Thomas Jesudason Isaac, Arun John Zechariah, Samuel N. J. David, Dass Prakash Yesupatham, Aparna Irodi, Vijay Aruldas, Shyamkumar Nidugala Keshava, Anand Zachariah, Gagandeep Kang, Joy John Mammen

**Affiliations:** a Department of Radiation Oncology, Christian Medical College Vellore, Vellore, India.; b Department of Forensic Medicine and Toxicology, Christian Medical College Vellore, Vellore, India.; c Department of General Medicine, Christian Medical College Vellore, Vellore, India.; d Department of Rural Unit for Health and Social Affairs, Christian Medical College Vellore, Vellore, India.; e Department of Palliative Medicine, Christian Medical College Vellore, Vellore, India.; f Department of Paediatric Nursing, College of Nursing, Christian Medical College Vellore, Vellore, India.; g Nalam Medical Centre and Hospital, Vellore, India.; h Best Scans & Lab, Vellore, India.; i Department of Pulmonary Medicine, Christian Medical College Vellore, Vellore, India.; j Distance Education Unit, Christian Medical College Vellore, Vellore, India.; k Department of Hospital Management Studies and Staff Training and Development, Christian Medical College Vellore, Vellore, India.; l Department of Transfusion Medicine, Christian Medical College Vellore, Vellore, India.; m Department of Radiodiagnosis, Christian Medical College Vellore, Vellore, India.; n OPD Services and Department of Medical Records, Christian Medical College Vellore, Vellore, India.; o Department of Interventional Radiology, Christian Medical College Vellore, Vellore, India.; p The Wellcome Trust Research Laboratory, Division of Gastrointestinal Sciences, Christian Medical College Vellore, Vellore, India.

## Abstract

**Background:**

In April 2021, during the peak of the second wave of the COVID-19 pandemic in India, hospitals overflowed with COVID-19 patients, and people hesitated to seek necessary care due to fear of contracting the disease. The UDHAVI helpline was set up by a tertiary care hospital in Vellore with the help of district administration, nongovernmental organizations, and various supporting agencies to provide general information, medical advice, counseling, and logistics support to the community.

**Methods:**

This is a retrospective study of all the phone calls made to the UDHAVI helpline between mid-May and mid-June 2021 during the second wave of the COVID-19 pandemic. The calls were electronically captured as part of the process, and the information was subsequently retrieved and analyzed.

**Results:**

In all, 677 calls were received. The lines for general information, medical advice, counseling, and logistics support received 168 (25%), 377 (56%), 15 (2%), and 117 (17%) calls, respectively. Home care kits, oxygen concentrators, and food were delivered by volunteers from local nongovernmental organizations and hospitals.

**Conclusion:**

We believe the details of our experience would be useful in the preparedness and mobilization of resources in the event of any public health emergency. As a result of this initiative, we propose an integrated partnership model for emergency response to any pandemic situation.

## Abstract (precis)

The authors describe how a tertiary care institution in India initiated a helpline during the second wave of the COVID-19 pandemic that leveraged institutional medical expertise and community health networks to better coordinate pandemic response efforts.

## Teaser

**Key Findings:**

- The UDHAVI helpline was developed to leverage medical and community health expertise to better coordinate pandemic response efforts that were hampered by resource constraints.
- The helpline raised callers' awareness of government quarantine guidelines, gave information on vaccination and testing, provided medical advice, offered psychological support and counseling to allay fears, and arranged transport of patients and supplies.
- The majority of helpline callers requested medical advice, including on vaccinations, pregnancy, COVID-19 transmission, risks, and drug interactions. The counseling line received the fewest calls.
- The outcome of the helpline initiative is an integrated partnership model for emergency response that can be used in the event of any public health emergency.

**Key Implication:**

- Our experiences in helpline establishment and management would be helpful to health care facilities should they choose to follow the UDHAVI helpline set-up process or improve the capacity of an existing helpline as an integrated partnership model for future community response to pandemic or endemic situations.

## BACKGROUND

Between 2011 and 2016, the World Health Organization (WHO) reported more than 1,000 epidemics across 168 countries. Previous public health crises have highlighted the risks of infectious disease outbreaks globally while illustrating the need for pandemic preparedness. Meanwhile, the scale and complexity of humanitarian emergencies continue to expand.^1^ In March 2020, the WHO officially declared COVID-19 a pandemic. Since then, COVID-19 has globally impacted health, politics, and the economy with catastrophic effects. In India, apart from the immense burden imposed on health care systems, people's daily lives were disrupted as the country adopted a partial to complete lockdown in the states to tackle COVID-19. The government recommended staying at home as much as possible, imposed strict quarantine for those who were exposed, and imposed isolation for those who were infected.^2^ During the peak of the first wave of the pandemic in September 2020, hospitals overflowed with patients, and people hesitated to seek necessary care due to the fear of contracting the disease.^3^^,^^4^ Additionally, numerous COVID-19-positive asymptomatic patients who were isolated at home faced emotional turmoil without psychosocial support from the community. The sharp increase in the number of infected people led to overcrowding in hospitals, and the shortage of medicines and oxygen created fear in the community.^5^^–^^7^ Additionally, misinformation regarding the disease, management, treatments, and protocols led to further anxiety and confusion in the community.^8^^–^^10^

In response to the pandemic at a local level, our hospital, a 3,000-bed tertiary care center in Vellore, India, serving about 8,000 outpatients per day, underwent a major reorganization of how the hospital functioned. A task force group for COVID-19 management was formed. The task force met daily and oversaw a significant overhaul of the hospital's infrastructure. First, a triage unit called the COVID Command Centre was set up for managing patients with COVID-19 and those suspected to have it. Second, the hospital suspended all elective hospital procedures and outpatient clinics and opened a fever clinic to screen individuals with suspected COVID-19 infection and manage them accordingly. Third, the intensive care unit capacity was increased, and a new wing was opened at the new hospital campus that was exclusively for COVID-19 patients.

Although these changes were crucial in response to the pandemic at the hospital level, a shift in focus was needed for a rapid and targeted response at the community level, especially during the second COVID-19 wave that hit India in April 2021.^11^^–^^13^ It became increasingly evident that the focus should address humanitarian needs and disease risk and include an emergency response team that was ready and had the capacity to respond.^14^ Because the public trusted the medical advice provided by our institution, even during non-emergency situations, the local administration and the public requested that the hospital create a medium for providing accurate medical advice that could help reduce panic, reduce hospital overcrowding, and allay community fears during the pandemic. In response, we developed an institutional response helpline called UDHAVI, which means “help” in Tamil, for the city of Vellore.

One of the crucial interventions in any public health emergency is to proactively communicate with the community about what is known and not known about the disease or adverse events. Evidence-based treatment protocols help save lives and minimize adverse consequences.^15^ The concept of using hotlines in public health crises is not new; it has been reported as 1 of the most used tools by WHO during the COVID-19 pandemic.^16^ These hotlines prove beneficial to allay some fears or mobilize community participation, especially when the health care infrastructure is stretched beyond its limits, but the desire as a community is to help in all ways possible.

The UDHAVI helpline was based on similar models that were rolled out from Delhi, India's capital, in response to the overwhelming load on local hospitals during the pandemic peak in April 2020, which was much earlier than in South India, where our institution is located.^17^^–^^21^ We created UDHAVI to establish a direct link between emergency responders and the general population to address the public's perceptions and concerns, communicate accurate information regarding the disease and vaccination, and raise awareness about the current government guidelines. The helpline also aimed to provide counseling, advice regarding health issues, and referral to other support services.^14^ We share our experiences on UDHAVI's formation process and report on its function and utility that may serve as a model for other health care facilities in future community responses to pandemic or endemic situations.

We created the UDHAVI helpline to establish a direct link between emergency responders and the general population to address the public's concerns and fears, lack of information, and lack of awareness about government COVID-19 guidelines.

## UDHAVI TEAM FRAMEWORK

**FIGURE 1:**
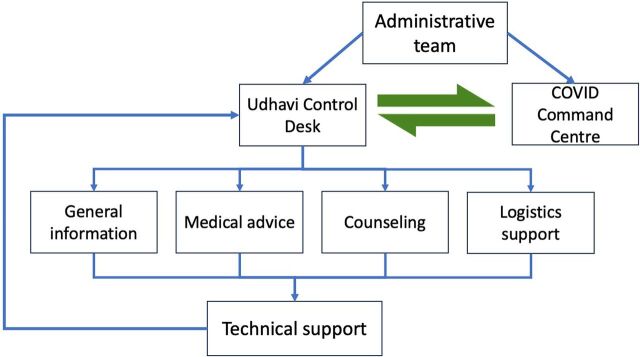
Framework and Team Hierarchy of the UDHAVI Helpline for COVID-19 Support in Vellore, India

The UDHAVI helpline was supported, managed, and staffed by an administrative team, a COVID Command Centre, a multidisciplinary team of volunteers, and a technical support team (Figure 1).

The administrative team garnered support for each of the 4 content areas (general information, medical advice, counseling, and logistics support) from volunteers among the hospital's medical faculty, nonmedical personnel, and support staff. The team was headed by the hospital's director and supported by representative administrators from the offices of the medical superintendent, general superintendent, and nursing services.

The hospital's COVID Command Centre functioned as the control desk and was managed by a multidisciplinary team of doctors, nonclinical personnel, and support staff. The hospital's clinical team that managed COVID-19 patients prepared protocols for home isolation and monitoring based on the existing scientific evidence. The control desk shared the home isolation protocol and instructions, including video links about how to use pulse oximeters and thermometers, with the asymptomatic and mildly symptomatic patients.

Each content area was staffed by a multidisciplinary volunteer team that comprised doctors, nonclinical personnel, and support staff. The administrative team circulated a sign-up sheet, and staff from departments that had stopped all non-essential services volunteered to staff the helpline and help with community outreach activities in Vellore. The volunteers were neither paid nor compensated by any other means. The hospital training department trained 75 doctors and 130 health care volunteers to be part of UDHAVI, including how to provide effective emergency responses. The training for each content area was handled by the respective team leaders. National and/or state guidelines and institutional guidelines were tailored to provide layperson-friendly advice by using audio recordings of simulated conversations.

The volunteers took turns answering the helpline calls in 3 shifts of 4 hours each over a period of 12 hours. If the calls were outside the 12-hour window, a callback was arranged via the COVID Command Centre.

**FIGURE 2:**
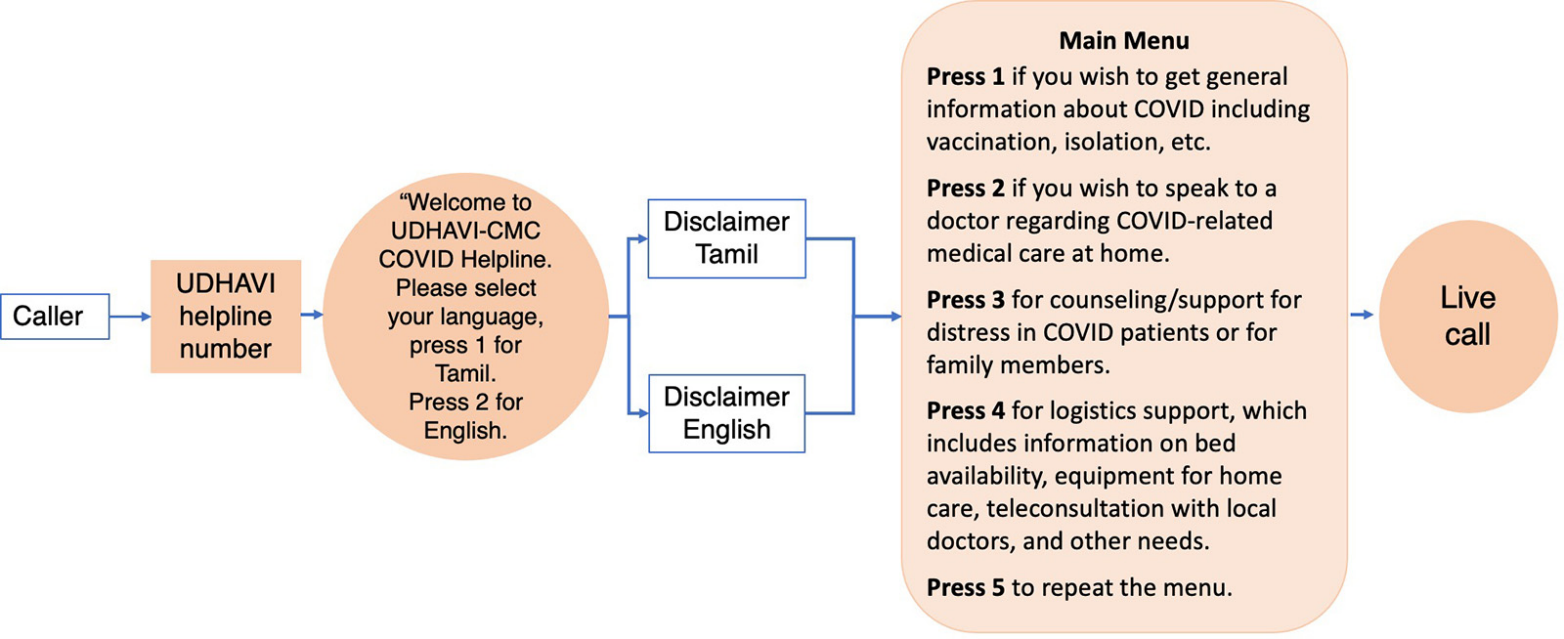
Outline of Interactive Voice Response for the UDHAVI Helpline for COVID-19 Support in Vellore, India Abbreviation: CMC, Christian Medical College Vellore.

The information technology personnel at the hospital provided technical support and were responsible for setting up and maintaining the interactive voice response (IVR) system through which patient calls were routed (Figure 2). Given the paucity of time and its ease of use, Exotel was chosen as the cloud telephony system to set up the virtual call support system to allow easy access to the public.^22^ This software supports multi-IVR call flows, automated call responses, automatic scaling when the number of calls surged, and call performance measures for both inbound and outbound calls. Exotel could be operated from both desktop and mobile platforms, and it provided features that allowed number masking for privacy. It also allowed for a voice menu system, which was made available in both Tamil and English. We employed double masking, which included number masking for both the caller and volunteers. A database was maintained with the volunteer contact numbers, and outbound calls were routed to a volunteer's mobile phone, depending on the option chosen by the caller on a round-robin basis. The first point of contact by the caller depended on their choice in the IVR. However, if the caller chose the wrong content area, the call was transferred to the most appropriate content area. This afforded a system where volunteers could activate and deactivate their phones depending on their availability without worrying about coverage for answering calls during any shift. A web dashboard provided administrators with access to call logs and reporting. This dashboard was built using JavaScript, HTML, and CSS with MongoDB database. Reports were generated daily from the dashboard. The technical support team also enabled the UDHAVI content area teams to communicate with each other via groups created within the WhatsApp application, which supported both instant messaging and voice-over Internet protocol service.

## UDHAVI HELPINE PROCESS

The UDHAVI helpline included 4 content areas: general information, medical advice, counseling, and logistics support. For each content area, the team leaders coordinated the workflow of their respective teams with volunteers within their group. The volunteers recorded the details pertaining to each caller in a Google Sheet. The details collected included call date and time; phone number, if callback was required; age, sex, and location; presence of influenza-like-illness symptoms and their duration; presence of warning signs and/or major comorbidities; COVID-19 test status; current level of care; recommended level of care; opinion was sought from the COVID-19 specialist consultant for medical advice, if any; and referral to logistics support helpline/Indian Medical Association (IMA) home monitoring. The information collected was reviewed by the volunteers, and they left additional remarks for follow-up if required.

### General Information

The general information area dealt with queries about COVID-19 that were not related directly to patient care or logistics support (such as vaccination and isolation guidelines). The team included volunteers who were from nonmedical disciplines within the hospital. The volunteers addressed doubts callers had about the disease, government guidelines with respect to movement and restrictions, clarifications about the vaccines, and locations of district vaccination centers. Separate information sheets for each category of query referenced the latest WHO, Government of India, and Tamil Nadu State Government guidelines. The updates also included information about the vaccination centers and lockdown restrictions based on government orders that were dynamically changing. To handle these dynamic changes, 2 subgroups were created to (1) update the information sheets daily and train the volunteers with the help of the staff training department and (2) handle the incoming calls. Due to the nature of the progression of the second wave, there was a significant difference between the information the group disseminated at the start and end of the UDHAVI initiative.

The general information area dealt with calls about COVID-19 that were not related to patient care or logistics support.

### Medical Advice

The medical advice area provided information on the care of COVID-19 patients at home, symptom assessment and management strategy, warning signs based on the information provided by the caller, and recommended level of care (hospitalization or home management). This area was managed by 2 levels of volunteer medical consultants. The level 1 consultant screened the patients by phone based on their history shared over the phone and gave advice based on the protocols. If the patient query could not be satisfactorily managed by a level 1 consultant or needed a focused expert opinion, the volunteer passed the information to the UDHAVI control desk, which then arranged a callback from a level 2 COVID-19 specialist consultant.

### Counseling

**FIGURE 3:**
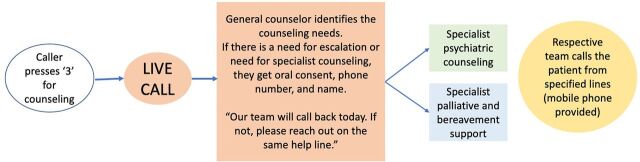
Workflow for the Counseling Area of the UDHAVI Helpline for COVID-19 Support in Vellore, India

The counseling area volunteers included nurses, doctors, chaplains, and psychologists. This area aimed to support and provide counseling to both COVID-19 patients and their family members who felt fearful, anxious, lonely, helpless, or had other psychological needs or distress. Patients or family members could access this area directly, or volunteers from other content areas could direct patients or family members who would benefit from counseling support to the counseling area. If the volunteer who supplied counseling support identified a greater need for specialist support or signs that indicated the need for further attention, they asked the caller if they would like to get additional help from a specialist, asked for their consent, and obtained their contact information. The specialist team would contact the caller later (Figure 3).

### Logistics Support

The logistics support area provided information to patients and their families on the importance of home isolation; the use of devices (e.g., oxygen concentrators, pulse oximeters, and thermometers) to monitor and care for themselves at home; and what to do if the situation deteriorated or did not improve within a prescribed period. The volunteers were nonmedical or paramedical staff and delivered home isolation kits to patients in and around Vellore, supplied food for the family in home isolation, arranged transportation of sick patients to hospitals, assessed patients discharged from the ward, and, if necessary, provided them with devices for use at home. The patient's health status was regularly monitored by follow-up. The patients returned the shared devices when they were no longer needed so they could be used for others in the community after appropriate disinfection procedures. The logistics support team kept an inventory of the devices and ensured that they were returned to circulation as quickly as needed within the community.

## METHODS

We conducted a retrospective study of all the phone calls made to the UDHAVI helpline between May 19 and June 18, 2021, during the second wave of the COVID-19 pandemic in Tamil Nadu, India.^21^

### Data Collection and Analysis

Quantitative and qualitative evaluation of UDHAVI performance occurred on a daily and weekly basis through online meetings with various stakeholders and helpline volunteers. Assessed variables included number of calls, location of the caller, language in which help was asked, nature of information needed, and duration of calls. At the daily debriefing meetings, the volunteers discussed the nature of questions asked and the information they needed help in retrieving and answering the queries. This information was used to add and refine the information sheets for the next day. After each review, the protocols or the workflow were changed to meet the needs of the community to streamline the process and improve efficiency. Ideas, suggestions, and feedback from the internal team members, helpline callers, and external collaborators were encouraged to improve the helpline. This continuous feedback from the community and other stakeholders contributed to identifying the gaps in the functioning of UDHAVI.

### Ethical Approval

The study was performed in accordance with the ethical standards in the 1964 Declaration of Helsinki and approved by the Christian Medical College Vellore institutional review board and ethics committee. Informed consent was waived due to the retrospective nature of the reporting, with no risk to human subjects involved.

## RESULTS

**FIGURE 4:**
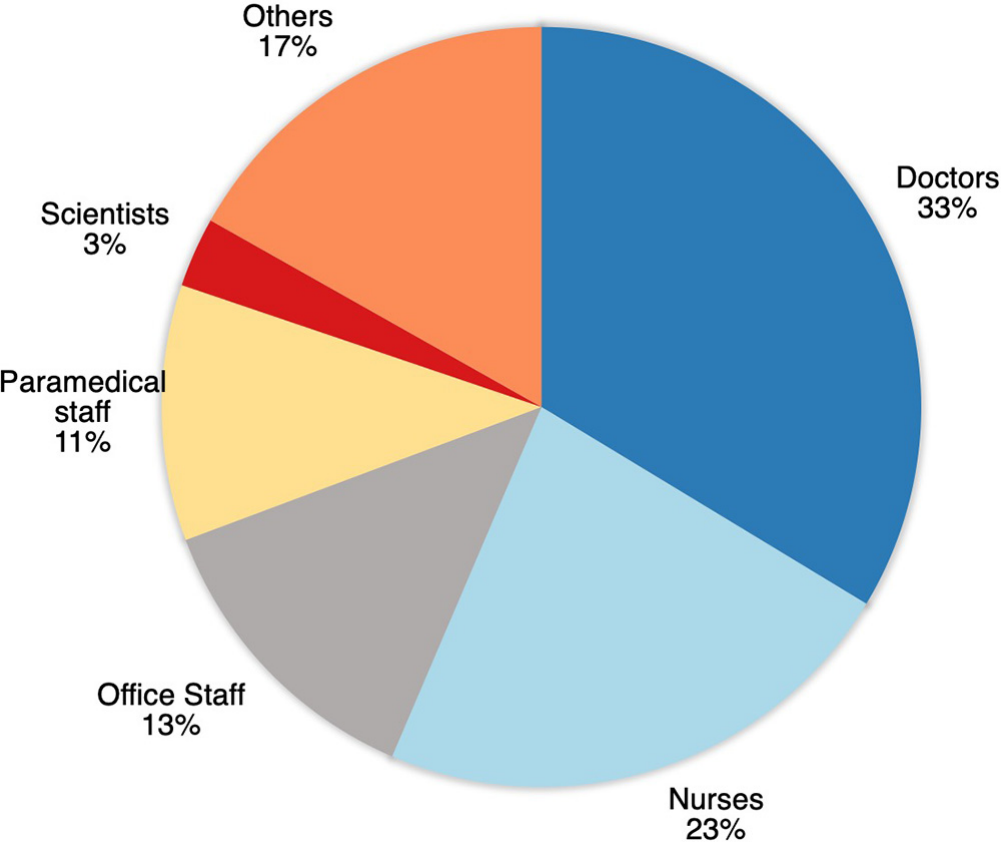
Composition of the Volunteer Team for the UDHAVI Helpline for COVID-19 Support in Vellore, India

A total of 101 trained volunteers from multiple health cadres and disciplines participated in the UDHAVI initiative (Figure 4). The “Others” category included members from the community or research staff.

During the study period, the helpline received a total of 677 calls to different content areas (Table 1). Most of the calls (56%) were related to seeking medical advice, which included queries about vaccinations, pregnancy, COVID-19 transmission, risks, drug interactions, etc.

**TABLE 1.** Summary of Calls Received by the UDHAVI Helpline for COVID-19 Support in Vellore, India

	General Information, No. (%)	Medical Advice, No. (%)	Counseling, No. (%)	Logistics Support, No. (%)
Total calls (N=667)	168 (25)	377 (56)	15 (2)	117 (17)
Most common query fielded	Vaccine related, 81 (48)	Level 1 calls, 181 (48)	-	Bed availability, 68 (58)
District of caller, Vellore	90 (54)	133 (35)	-	63 (54)
State of caller, Tamil Nadu	152 (91)	335 (89)	-	112 (96)
Average calls per day	6	13	-	4

### General Information

The volunteers in this area received a total of 168 calls. Most calls (91%) were from Tamil Nadu, followed by Andhra Pradesh (3%); West Bengal (2%); Jharkhand (1%); and Bihar, Karnataka, and Puducherry (3%). The most common query was related to vaccination (48%), but volunteers also received queries about bed availability (13%), quarantine (8%), testing for COVID-19 (7%), and miscellaneous issues (2%). Fifteen percent of the callers needed further advice from a medical practitioner and were redirected to the medical advice area.

### Medical Advice

This area received a total of 377 calls, of which 89% were from Tamil Nadu. Few calls were received from other parts of India, including 4% from Andhra Pradesh; 1% each from Jharkhand, Karnataka, and West Bengal; and less than 1% from Uttar Pradesh, Puducherry, Odisha, Meghalaya, Chhattisgarh, Kerala, and Bihar.

The median age of those who sought medical advice was 44 years (interquartile range: 33–59). The majority were male (58%). Almost half the callers (49%) reported to be COVID-19 positive, 7% reported a negative status, and 28% had not tested for the infection. Of the 377 callers, 181 (48%) required Level 1 care (ward-based care involving support and added advice from the critical care team); 61 (16%) were recommended Level 2 care (admission requiring more specific intervention or observation with support required for single failing organ system); and 135 (36%) did not need any hospitalization. Additionally, 193 (51%) reported having influenza-like-illness symptoms, 98 (26%) had comorbidities, and 83 (22%) had warning signs for which they were recommended immediate hospital care. Sixty-five patients (17%) were referred to the logistics support area for additional support.

### Counseling

The counseling area received a total of 15 calls either directly or via referral from other areas. The volunteers spoke to the patient or their relatives to allay their concerns. No further referrals were made to psychiatrists.

### Logistics Support

The logistics support area received 117 (17%) of the total calls to the helpline. More than half of these queries (58%) were related to the availability of beds in the hospital or intensive care unit. Other queries included requests for ambulance service (7%) and oxygen concentrators (5%), and 16% of the callers had general inquiries that were answered and not redirected to other areas. Through the helpline, the logistics team also made deliveries to local residents (Table 2).

**TABLE 2.** Type of Delivery Made by UDHAVI Logistics Team for COVID-19 Support in Vellore, India

Type of Delivery	Total No. Delivered
Home care kits	10
3 meals per day per supported persons (no. of days)	14 (20 days)
Oxygen concentrator (1.5 L)	14
Oxygen concentrator (10 L)	3

## LESSONS LEARNED

We report the technical aspects of setting up a pandemic response helpline through the collective effort of a medical college with district administration and local support to provide medical advice, surveillance, and material support during the second wave of the COVID-19 pandemic. UDHAVI was conceptualized as a call to respond to the unprecedented crisis of the patients within the city of Vellore. The lessons learned from this helpline were used in managing the subsequent COVID-19 pandemic wave by the institution and the larger collective network in Vellore. However, we believe that the details of our helpline management would be useful in the preparedness and mobilization of resources in response to any public health emergency.

### Framework Establishment

Although there were helpline models available in Delhi and Tamil Nadu, we felt there was still space for the UDHAVI helpline to be relevant and useful. We wanted to leverage our medical and community health expertise to help coordinate efforts within our resource-constrained city for patients seeking care during the pandemic. We made efforts to ensure that UDHAVI incorporated the following guidelines from the WHO emergency response framework to provide support to and meet the needs of the community.^1^

- Principle of humanity—saving lives and relief of suffering
- Community focus, community engagement
- Evidence-based interventions—program planning and implementation
- Partnership with key stakeholders
- Training and education
- Accountability—evaluation and feedback

### Human Resources Management

During the pandemic, management of human resources was an essential task that required a thoughtful and adaptable approach. Although it was important for us as a hospital to act as responders to community needs, we also had a responsibility to ensure the well-being of our hospital employees. Through regular emails and virtual meetings, we clearly communicated to employees about the pandemic situation, safety measures, and changes in policy. We also encouraged and supported vaccination efforts among employees by providing them with information about the vaccination locations and appointment times. We believed that once the employees felt supported and had reliable information, there was enough traction for administrative teams to request volunteers for the pandemic response. This allowed us to establish the COVID Command Centre, which comprised the pandemic response team and the UDHAVI helpline.

The UDHAVI teams were also sensitive to the fact that the helpline volunteers were health care professionals or individuals working in the health care sector who were also living through the same traumatic events as their callers, raising the risk of double exposure to trauma and experiencing helplessness and burnout.^23^^–^^25^ The volunteers reported that the team evaluations, feedback, counseling, and coordination among the teams from different areas helped them feel supported through the experience while making an impact.

### Leadership and Training

During the COVID-19 emergency, our institution explicitly carried out critical functions detailed in the WHO incident management system, including leadership, partner coordination, information and planning, health operation, technical expertise, operations support, logistics, finance, and administration. The leadership was provided by the representatives from the administration team who worked with the district public administration, providing information on the COVID-19 guidelines released periodically by the state government.^26^^,^^27^

The preparation phase of UDHAVI took 2 weeks, during which evidence-based guidelines and protocols were developed. Representatives from the administrative team decided to include 4 areas in the helpline and helped identify the leaders for each area. Volunteers then received training in each of the areas. The training provided by the hospital to the volunteers ensured that all callers received validated medical advice. This was important at the time, as India contributed to about 16% of the misinformation on COVID-19 worldwide that was circulated through social media or Internet-based sources.^8^^–^^10^ Mock sessions contributed to increasing confidence in the delivery of appropriate services and familiarity with using the apps and electronic devices. Medical advice was identified as the priority need for the community during the pandemic. Hence, we consider a milestone of the UDHAVI initiative to be that volunteers shared scientific, validated, and evidence-based information that helped reduce confusion and fear in the community.

We consider a milestone of the UDHAVI initiative to be that volunteers shared scientific, validated, and evidence-based information that helped reduce confusion and fear in the community.

### Community Mobilization and Scale-Up

Though originally conceived as a hospital-driven initiative, UDHAVI grew to become a community-based effort with support from local nongovernmental organizations, including Hope House, Seb's Vellore, Vellore Institute of Technology Alumni, and Pisces Wellness and Health, as well as professional bodies such as the IMA local chapter.^28^^–^^30^ Before the UDHAVI initiative was implemented, Christian Medical College Vellore and IMA Vellore conducted a continuing medical education program to ensure the use of evidence-based protocols for COVID-19 management at the hospital. By leveraging this connection and volunteers through the IMA network and the community, we formed the core group that engaged in meeting the needs of the community through the different areas. The hospital's Community Health Department and Low-Cost Effective Care Unit conducted community outreach programs, and the Rural Unit for Health and Social Affairs managed the home monitoring and support services in their coverage areas with logistic and advisory support through UDHAVI. The IMA partnered with UDHAVI due to the synergy with their efforts in Vellore and followed up home-isolated patients with mild symptoms through daily callbacks. The interventions were done with appropriate technical help and operational support after careful planning and continued updating. Nongovernmental organizations were selected to partner with UDHAVI as their efforts complemented the UDHAVI logistics support area in its outreach during the pandemic (e.g., providing food and transporting oxygen concentrators). These partnerships strengthened the capacities and skills of the staff and enhanced the response and functioning of UDHAVI.^31^ In the subsequent COVID-19 waves, we were able to deploy the helpline with a quick turnaround time.

Although we understood the importance of counseling during the pandemic when we developed the helpline, the data from the counseling area did not show this. The counseling area received the least number of direct or referred calls, indicating that this area may not have been not a priority recognized by the public. The data may also suggest that callers did not fully understand the scope of service; proactively seeking psychological help and support is still relatively new to the Indian culture.^32^^,^^33^ This does not understate the public's need for psychological support during this time; however, as the call data showed, the public's immediate need and priority was medical care.

While setting up the helpline, we realized that the logistics support area might be more localized; however, the other areas for general advice, counseling, and specific medical advice could assist those in other parts of India, as well. This allowed us to help callers from outside the district or state with queries related but not limited to bed availability in our hospital, reassurance about taking the vaccine, and when to seek hospitalization. We ensured that our protocols were evidence based and in compliance with the national and state guidelines, and flexibility in the protocol development easily ensured scalability and deployment. Scaling up required us to lean into our community and leverage existing partnerships that were well established during non-emergency periods. For example, it allowed us to direct callers from outside Vellore to IMA Vellore, which could then provide information about health facilities/helplines in their respective states/districts available through the IMA network.

### Helpline Promotion

It was critical to promote the helpline to ensure that individuals had access to information, support, and resources during the pandemic. To reach as many people as possible, we used various media, including websites, social media platforms, and local newspapers. We also created flyers in both English and Tamil that were displayed at all campuses of the hospital and its rural units.^34^^,^^35^

### Program Generalizability

**FIGURE 5:**
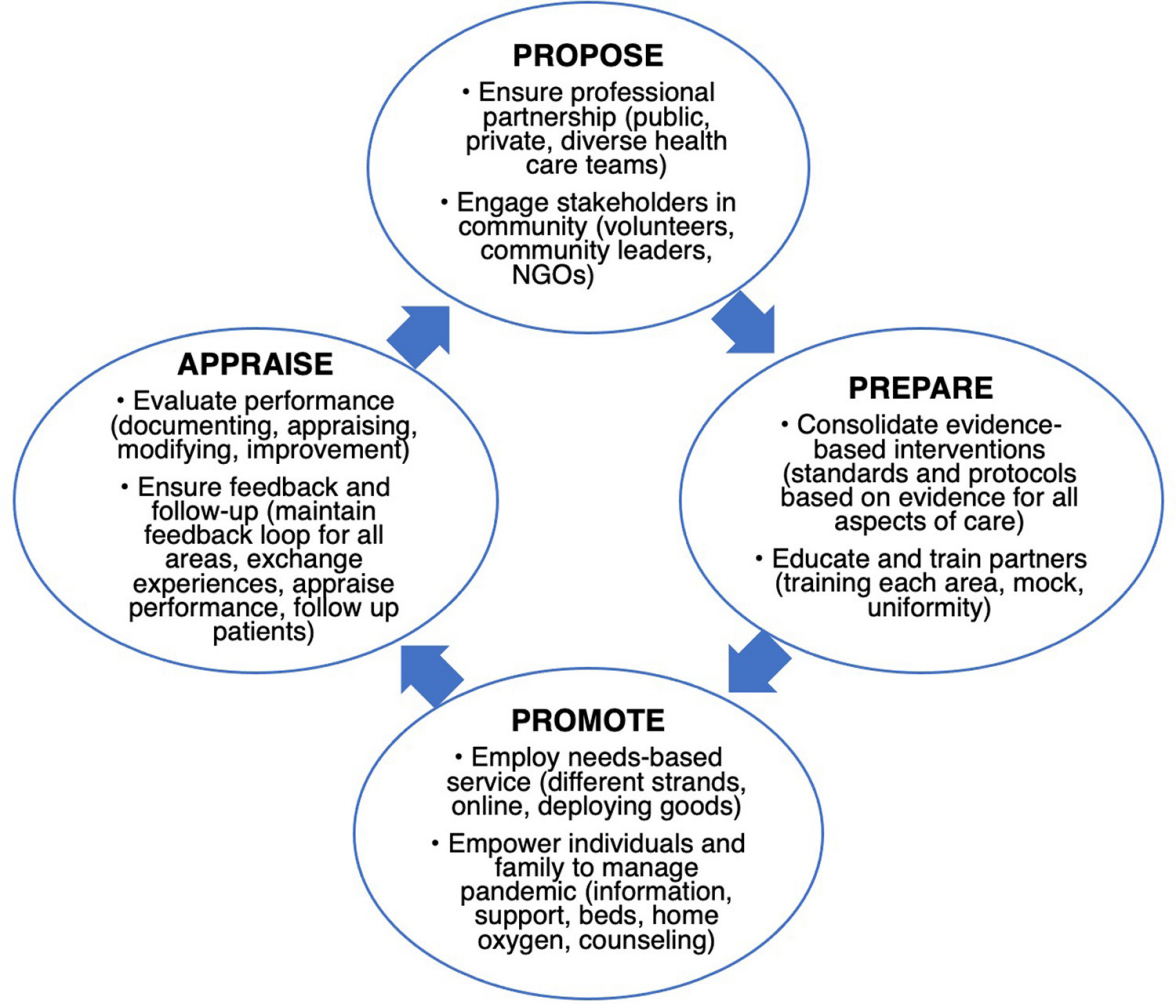
Integrated Partnership Model Based on the UDHAVI Helpline for COVID-19 Support in Vellore, India Abbreviation: NGO, nongovernmental organization.

We took 2 weeks to initiate UDHAVI during the peak of the second wave. In hindsight, the process could have been started earlier, but we faced challenges considering the unprecedented burden of the second wave on the institution. Based on our experience, we developed an integrated partnership model that could be used for future emergency response in any pandemic or epidemic. The model has 4 phases with 8 strategies (Figure 5).

## CONCLUSION

We shared our institutional experience of setting up a helpline during a pandemic so that health care facilities may either follow our set-up process or improve the capacity of an existing helpline with the aim of saving lives and improving health in the community. If an integrated partnership-based emergency response framework like UDHAVI is put into place by the institutions during a non-emergency period, deploying it during any public health emergency would be possible and timely.

## Notes

**Peer Reviewed**

**First published online:** October 20, 2023.

## References

[1] World Health Organization (WHO). *Emergency Response Framework (ERF)*. 2nd ed. WHO; 2017. Accessed October 2, 2023. https://apps.who.int/iris/handle/10665/258604

[2] Kumar SU, Kumar DT, Christopher BP, Doss CGP. The rise and impact of COVID-19 in India. Front Med (Lausanne). 2020;7:250. 10.3389/fmed.2020.00250. 32574338 PMC7256162

[3] Wong LE, Hawkins JE, Langness S, Murrell KL, Iris P, Sammann A. Where are all the patients? Addressing Covid-19 fear to encourage sick patients to seek emergency care. NEJM Catal Innov Care Deliv. May 14, 2020. Accessed October 12, 2023. https://catalyst.nejm.org/doi/full/10.1056/CAT.20.0193

[4] Bagcchi S. Stigma during the COVID-19 pandemic. Lancet Infect Dis. 2020;20(7):782. 10.1016/S1473-3099(20)30498-9. 32592670 PMC7314449

[5] Reuters. As COVID-19 floods India’s hospitals, the better-off also scramble for care. The Economic Times. April 29, 2021. Accessed October 2, 2023. https://economictimes.indiatimes.com/news/india/as-covid-19-floods-indias-hospitals-the-better-off-also-scramble-for-care/articleshow/82313815.cms

[6] Pandey V. Covid-19 in India: patients struggle at home as hospitals choke. BBC News. April 26, 2021. Accessed October 2, 2023. https://www.bbc.com/news/world-asia-india-56882167

[7] Dutta T. India’s hospitals choke under oxygen shortage amid raging pandemic. The National. April 23, 2021. Accessed October 2, 2023. https://www.thenationalnews.com/world/asia/india-s-hospitals-choke-under-oxygen-shortage-amid-raging-pandemic-1.1209602

[8] Badrinathan S. Opinion | India is facing an epidemic of misinformation alongside covid-19. Washington Post. June 7, 2021. Accessed October 2, 2023. https://www.washingtonpost.com/opinions/2021/06/07/india-misinformation-covid-19-pandemic

[9] Al-Zaman MS. Prevalence and source analysis of COVID-19 misinformation in 138 countries. IFLA J. 2022;48(1):189–204. 10.1177/03400352211041135

[10] Freckelton QC I. COVID-19: fear, quackery, false representations and the law. Int J Law Psychiatry. 2020;72:101611. 10.1016/j.ijlp.2020.101611. 32911444 PMC7351412

[11] Kumar S. Second wave of COVID-19: emergency situation in India. J Travel Med. 2021;28(7):taab082. 10.1093/jtm/taab082. 34037783 PMC8244620

[12] M SJ. Facing the second wave of COVID-19. The Hindu. December 31, 2021. Accessed October 2, 2023. https://www.thehindu.com/news/national/tamil-nadu/facing-the-second-wave-of-covid-19/article38074727.ece

[13] What to know about India’s coronavirus crisis. The New York Times. November 17, 2021. Accessed October 2, 2023. https://www.nytimes.com/article/india-coronavirus-cases-deaths.html

[14] Anand V, Verma L, Aggarwal A, Nanjundappa P, Rai H. COVID-19 and psychological distress: lessons for India. PLoS One. 2021;16(8):e0255683. 10.1371/journal.pone.0255683. 34347847 PMC8336880

[15] Emergency risk communication (ERC) five-step capacity-building package. World Health Organization. May 7, 2019. Accessed October 2, 2023. https://www.who.int/europe/multi-media/item/emergency-risk-communication-(erc)-five-step-capacity-building-package

[16] World Health Organization (WHO). Regional Office for Europe. *Setup and Management of COVID-19 Hotlines*. WHO Regional Office for Europe; 2020. Accessed October 2, 2023. https://apps.who.int/iris/handle/10665/336027

[17] Delhi Fights Corona. Accessed October 2, 2023. https://delhifightscorona.in

[18] Sharma N, Sharma P, Basu S, et al. Second wave of the COVID-19 pandemic in Delhi, India: high seroprevalence not a deterrent? Cureus. 2021;13(10):e19000. 10.7759/cureus.19000. 34853742 PMC8609204

[19] Delhi Corona. Accessed October 2, 2023. http://corona.delhi.gov.in

[20] Covid Management | District Southwest, Government of Delhi. Accessed October 2, 2023. https://dmsouthwest.delhi.gov.in/covid-management

[21] Government of India. Ministry of Health and Family Welfare. Accessed October 2, 2023. https://www.mohfw.gov.in

[22] Exotel. Accessed October 2, 2023. https://exotel.com

[23] Kulkarni A, Khasne RW, Dhakulkar BS, Mahajan HC. Burnout among healthcare workers during COVID-19 pandemic in India: results of a questionnaire-based survey. Indian J Crit Care Med. 2020;24(8):664–671. 10.5005/jp-journals-10071-23518. 33024372 PMC7519601

[24] Joshi G, Sharma G. Burnout: a risk factor amongst mental health professionals during COVID-19. Asian J Psychiatr. 2020;54:102300. 10.1016/j.ajp.2020.102300. 32683251 PMC7341036

[25] Chakma T, Thomas BE, Kohli S, et al. Psychosocial impact of COVID-19 pandemic on healthcare workers in India & their perceptions on the way forward - a qualitative study. Indian J Med Res. 2021;153(5& 6):637–648. 10.4103/ijmr.ijmr_2204_21. 34596596 PMC8555609

[26] Chandrababu D. Tamil Nadu brings more hands for Covid helpline as cases surge. Hindustan Times. April 19, 2021. Accessed October 2, 2023. https://www.hindustantimes.com/india-news/tamil-nadu-brings-more-hands-for-covid-helpline-as-cases-surge-101618837171994.html

[27] Prevent from Corona | Vellore District, Government of Tamil Nadu. Accessed October 2, 2023. https://vellore.nic.in/prevent-from-corona

[28] The Hope House. Accessed October 2, 2023. https://www.indiahopehouse.org

[29] Our people. Seb’s Projects India. Accessed October 2, 2023. https://www.sebsprojectsindia.org/our-people

[30] Pisces Health and Wellness Private Limited. Tofler. Accessed October 2, 2023. https://www.tofler.in/pisces-health-and-wellness-private-limited/company/U74999TZ2019PTC031773

[31] Narayanan V. Helpline for COVID-19 patients in Vellore, Ranipet, Tirupathur districts. The Hindu. June 3, 2021. Accessed October 2, 2023. https://www.thehindu.com/news/national/tamil-nadu/helpline-for-covid-19-patients-in-vellore-ranipet-tirupathur-districts/article34717036.ece

[32] Sanghvi PB, Mehrotra S. Help-seeking for mental health concerns: review of Indian research and emergent insights. J Health Res. 2022;36(3):428–441. 10.1108/JHR-02-2020-0040

[33] Srivastava K, Chatterjee K, Bhat P. Mental health awareness: the Indian scenario. Ind Psychiatry J. 2016;25(2):131–134. 10.4103/ipj.ipj_45_17. 28659690 PMC5479084

[34] Christian Medical College Vellore Facebook page. Announcing Udhavi – a free helpline offering information and support for people concerned about COVID-19, for residents of Vellore and neighbouring districts. Accessed October 2, 2023. https://www.facebook.com/cmcvelloreindia/posts/5773137996060014

[35] Udhavi: CMC COVID Helpline. Give CMC. May 22, 2021. Accessed October 2, 2023. https://givecmc.org/cmc-covid-helpline

